# Shared volatile organic compounds between camel metabolic products elicits strong *Stomoxys calcitrans* attraction

**DOI:** 10.1038/s41598-020-78495-9

**Published:** 2020-12-08

**Authors:** Merid Negash Getahun, Peter Ahuya, John Ngiela, Abel Orone, Daniel Masiga, Baldwyn Torto

**Affiliations:** 1 International Centre of Insect Physiology and Ecology (ICIPE), P.O. Box 30772‑00100, Nairobi, Kenya; 2 Present Address: Biotechnology Research Institute, Kenya Agricultural & Livestock Research Organization (KALRO), Nairobi, Kenya

**Keywords:** Chemical ecology, Metabolic pathways, Metals

## Abstract

The sources of animal odours are highly diverse, yet their ecological importance, in host–vector communication, remains unexplored. Here, using the camel (host)–*Stomoxys calcitrans* (vector) interaction, we collected and analyzed the Volatile Organic Compounds (VOCs) of camels from four of its different odour sources: breath, body (skin), urine, and dung. On non-metric model multivariate analyses of VOCs we show that substantial chemo-diversity exists between metabolic products associated with an individual camel. VOCs from the four metabolic products were distinct and widely segregated. Next, we show electrophysiologically, that VOCs shared between metabolic products activated more Olfactory Sensory Neurons (OSNs) and elicited strong behavioural attractive responses from *S. calcitrans* under field conditions independent of geography. In our extended studies on house flies, the behavioural response to these VOCs appears to be conserved. Overall, our results establish that VOCs from a range of metabolic products determine host–vector ecological interactions and may provide a more rigorous approach for discovery of unique and more potent attractants.

## Introduction

Stable fly (*Stomoxys calcitrans* Linnaeus, 1758) is a major vector of pathogens such as trypanosomes, anaplasma, and viruses of domestic animals^1–5^. Furthermore, *S. calcitrans* is highly anthropophilic and well adapted to various habitats, including the urban setting that makes it one of the most important vectors of domestic animals globally. *S. calcitrans* feeds on a wide range of both domestic and wild animals^2,6^. Other than the diseases they transmit the nuisance and stress caused by stable flies painful bites can reduce the productivity of livestock, by decreasing pasture time, investing in defensive behaviour, reduce weight gain and lead to suffering in animals and humans^7,8^. Livestock production systems such as dairy farms are seriously affected by stable flies and house flies as they are an ideal site for stable flies breeding and establishment. In the USA alone Stable flies induce a cost of 2.2 billion dollar annually in livestock business^8^ and furthermore, the same flies together with house flies developed resistance to multiple insecticides^9^ that have spurred the search for alternative vector management options. The recent data established for the genome of *S. calcitrans* has revealed that genes involved in chemoreception are highly abundant in *S. calcitrans*^10,11^, indicating that olfaction could play a significant role in the biology and ecology of this fly. Although the chemical ecology of oviposition behaviour of *S. calcitrans* is relatively well studied^12–15^ its olfactory-based interaction with a blood meal source is less understood.

Here, we aimed to investigate the chemical communication between *S. calcitrans* and the camel one of its highly preferred bloodmeal sources^2^. We examined the major sources of VOCs including breath^16,17^, body (sweat via skin)^18,19^ urine^20^ and feces^12,15,21,22^. We posited that, to what extent do odours emitted from these different metabolic products of the camel sources contribute to its overall smell and interaction with *S. calcitrans*. Hence, we analyzed and identified the odours from four camel metabolic products by Gas-Chromatography–Mass-Spectrometry (GC–MS). We identified both common and different volatile metabolites originated from different camel’s sources. Then, we measured the coding of VOCs by physiological responses of *S. calcitrans* olfactory sensory neurons using Single Sensillum Recording (SSR). Finally, we correlated the OSNs coding with behavioural responses to selected VOCs identified from the four camel’s odour sources to decipher the logic of chemical communication between *S. calcitrans* and camel.

Our results provide the first study which show different chemical signature of VOCs released by four different metabolic products of camel and, their strong interaction with olfactory sensory neurons of the stable fly, with comparative behavioural attractions.

## Results

### VOCs constitutes of the four camel’s metabolic products

**Figure 1:**
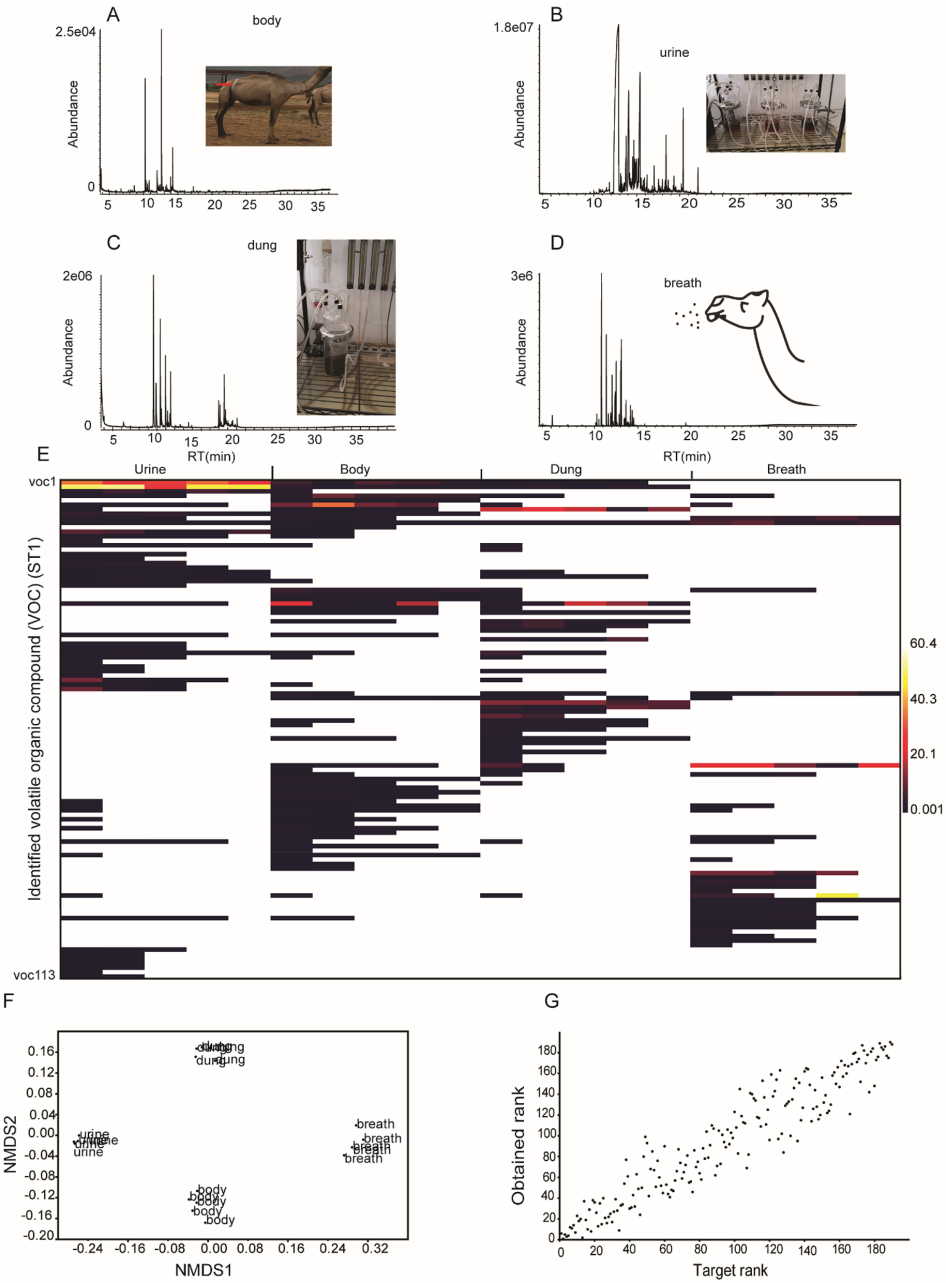
GC–MS profiles showing VOCs detected in the four metabolic products of camel (n = 5 camels). (**A**–**D**) GC–MS traces obtained from body, urine, dung and breath, respectively. (**E**) Heatmap coded matrix showing relative percent contribution of individual VOCs to the total composition of each metabolic product; urine, body, dung, and breath. (**F**) Multivariate analysis of camel VOCs identified from the four metabolic products by non-metric multidimensional scaling (NMDS). (**G**) The graph shows the goodness of fit measure for points in non-metric multidimensional scaling, with a stress value of 0.1619, Bray–Curtis.

GC–MS analysis identified 113 volatile organic compounds from the four camel’s odour sources; dung, body, urine, and breath, which belong to a diverse class of organic chemicals (Fig. 1A–E, ST1). These four sources share several VOCs qualitatively (Fig. 1A–E, Sup Table 1). The relative abundances of these VOCs were used to generate a matrix across the four metabolic products (Fig. 1E, Sup Table 1). The obtained volatilome varied in both quantity and qualitatively (Fig. 1F) with stress value of 0.16, showing the better fit for the dissimilarity (Fig. 1G). Each metabolic product was characterized by a distinct pattern of VOCs, ANOSIM analysis showed a significant difference between the four odour sources in their VOCs, P = 0.0001, R = 0.9807. Terpenes and aromatic hydrocarbons defined the odour of breath, whereas body odour was defined by the aldehydes, nonanal and decanal. Urine odour was defined by the phenolic compounds *p*-cresol, ethyl-3 phenol, and 4-propylphenol and the monoterpenes α- and β-pinene defining the odour of dung.

The GC–MS analysis demonstrated that *p*-cymene might be the only VOC conserved in all the four metabolic products with detectable quantitative differences (Fig. 1E, “Sup Table”).

### The physiological response of *S. calcitrans* OSNs to camel derived semiochemicals

**Figure 2:**
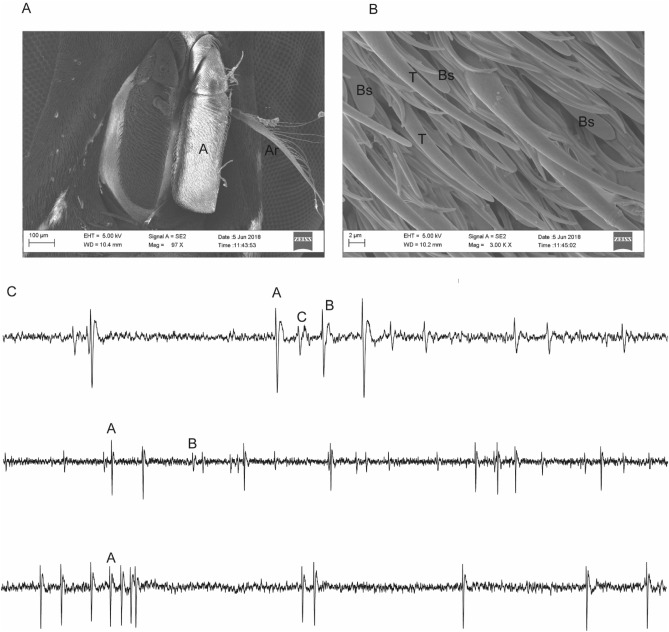
*S. calcitrans* antennal morphology and sensory neurons distribution. (**A**) Indicate the third antennal segment (Ar arista). (**B**) A scanning electron micrograph of the antenna of *S. calcitrans,* showing the olfactory sensilla micrographs of basiconic (Bs) and trichoid (T) sensilla. (C) Selected Single sensillum recording traces showing the number of OSNs per sensillum, top shows three OSNs per sensillum, middle two and the bottom shows single OSN per sensillum.

Having identified descriptive volatiles of each metabolic product, next we analyzed 31 selected compounds for physiological response by *S. calcitrans* using single sensillum recoding techniques at 10^−3^ v/v dilution. We selected odourants that were unique and conserved between the four metabolic products based on their availability. Electrophysiological recording was done only from basiconic sensilla (Fig. 2A,B). The olfactory sensory neurons on the antenna consistently showed spontaneous activity. From the targeted sensilla (n = 21), most targeted sensilla housed one to three OSNs per sensillum based on their spike amplitude (Fig. 2C).

### Coding of camel derived semiochemicals by *S. calcitrans* OSNs

**Figure 3:**
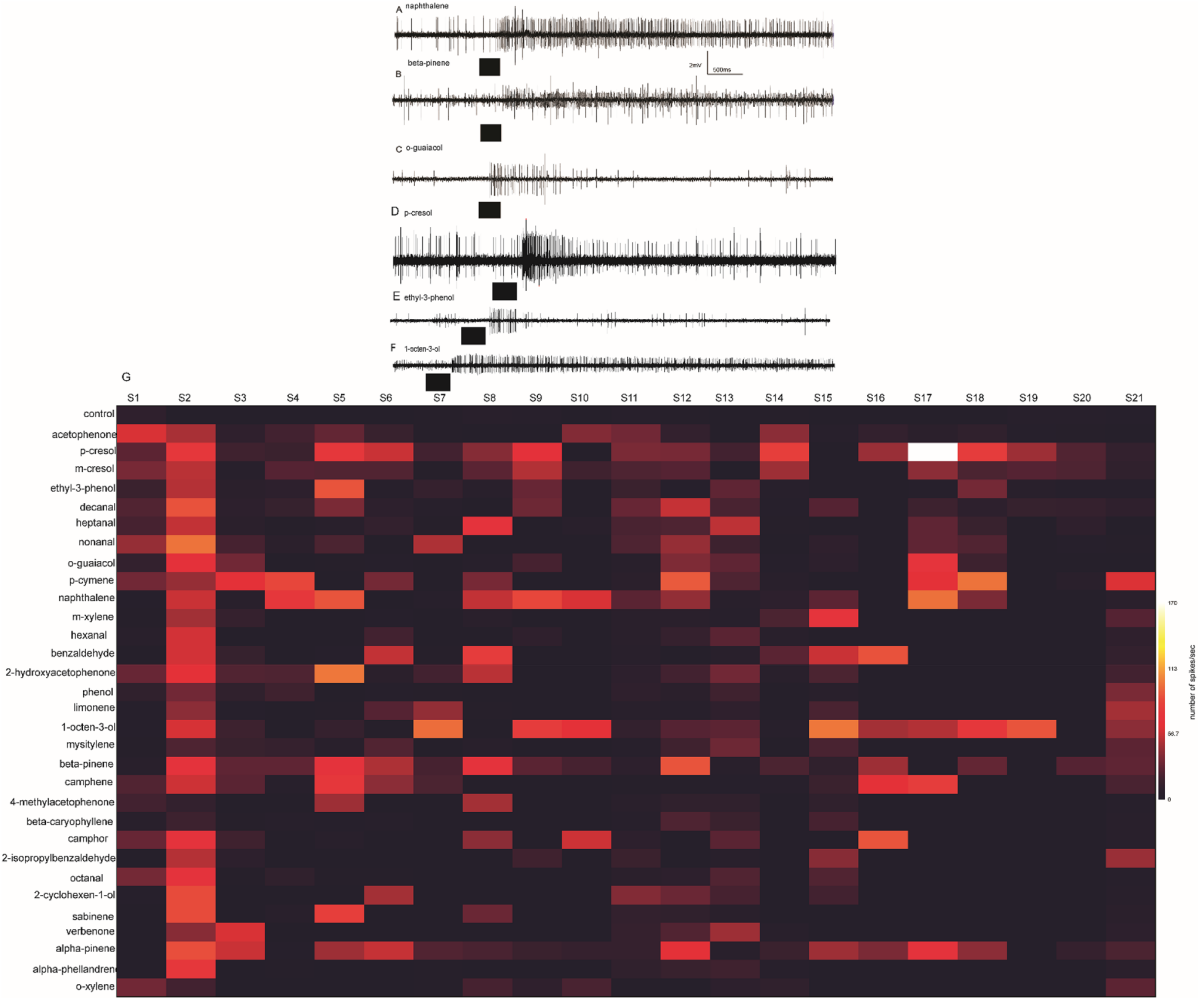
Olfactory coding of camel derived volatile chemicals*.* (**A**–**F**) show representative traces of OSNs of *S. calcitrans* response to naphthalene, β-pinene, guiacol, p-cresol, ethyl-3-phenol, and 1-octen-3-ol, respectively. Black box indicates 500 ms odour stimulation*.* (**G**) Shows the matrix coding based on the number spikes/sec elicited by 21 sensilla to the 31 camel derived compounds (left) and control.

We characterized the response of olfactory sensory neurons (OSNs) housed in basiconic sensilla of the stable fly to the 31 selected compounds. The responses from targeted sensilla, against tested compound OSNs interactions were found to be different. For example, in (Fig. 3A,B). B-neurons (Fig. 3A) and C-neurons (Fig. 3B) in the two representative sensilla increased their spike frequency when stimulated with naphthalene and β-pinene, while the spike frequency of A neuron in Fig. 3A and A and B neurons in Fig. 3B were not affected, showing the selectivity of co-localized OSN receptor for the given odour in the same sensillum. In another sensillum the A neuron was activated but B neurons remained unaffected (Fig. 3C–E). Notably, there was variation in background spontaneous activity of the different neurons per a given sensillum, as observed for A neurons spikes which are very rare as compared to the colocalized B neurons (Fig. 3A).

**Figure 4:**
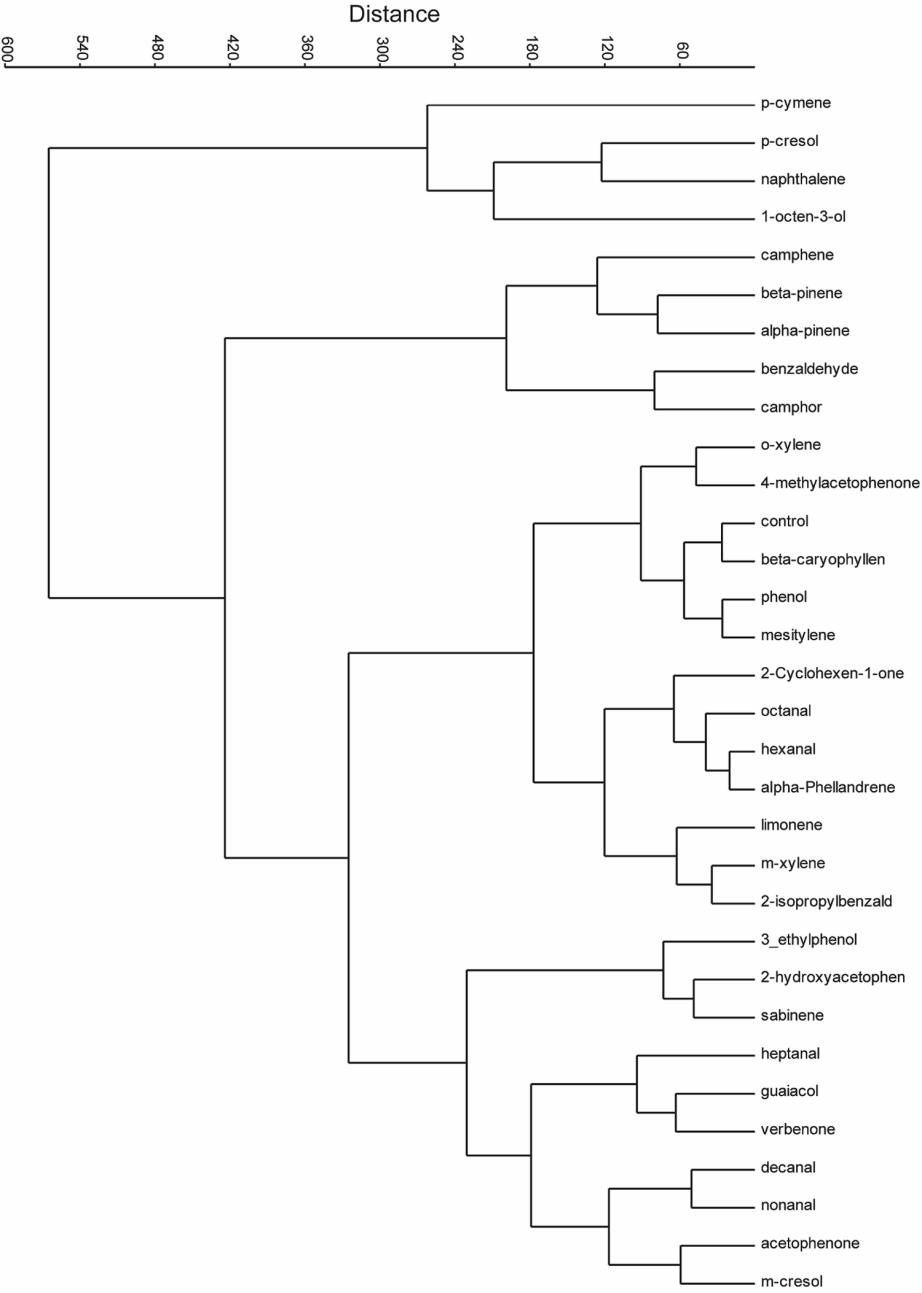
Cluster analysis of 21 basiconic sensilla based on their response profile, each tested with 31 odorants. The classification was carried out with Ward’s method.

Physiological response variation between OSNs were observed. Each cell in the heatmap (Fig. 3G) represents the number of spikes of a OSN (after subtracting the spontaneous activity before stimulation). The number of neurons housed in the contacted sensillum varies from 1 to 3 (Fig. 2), no OSN pinching was observed, due to intense firing when stimulated, as is the case in Drosophila which makes the responding OSN differentiation based on amplitude difficult to ascertain, as the OSN amplitude reduced, were one forced to pool the number of spikes. From the response profile some OSNs were highly selective responding to only a few compounds per sensillum out of the 30+ compounds screened with 10^−3^ v/v dilution (Fig. 3G). Others responded to several compounds, and broadly tuned (Fig. 3G). A good example for broadly tuned OSN is S3 in (Fig. 3G), and more selective is S1, and S20 (Fig. 3G). Furthermore, some compounds elicited a sustained response (Fig. 3A,B,F), whereas most of the responses were phasic (Fig. 3C,E).The activation pattern of each VOC to the targeted OSNs also varied, for example*,* p-cymene which is found in all four metabolic products activated 52%, whereas *p*-cresol which is shared between urine, dung and body odours, activated 67% of the targeted OSNs. Naphthalene, a shared VOC between body and breath odours activated 52% of the targeted OSNs. Similarly, α-pinene, shared between body, dung and urine odours activated 52% of OSNs. However, 1-octen-3-ol detected only in body odour, activated 61% of the OSNs. The VOCs decanal, heptanal, hexanal, detected only in body, and 2-cyclohexen-1-one, 2,6-dimethylphenol, 2-hydroxyacetophenone, detected only in urine odour activated up to 48% of the targeted OSNs (Fig. 3G). The interaction between odour and OSN resulted in a response which represented a specific olfactory adaptation. The maximum spikes elicited observed from the tested odours was 169 spikes/sec by 10^−3^ v/v *p*-cresol stimulation from sensilla 17 (S17) (Fig. 3G). A cluster analysis of all activated OSNs based on their response patterns to the 31 compounds from the four camel metabolic products revealed that the Stable fly OSNs response pattern classified into different clusters (Fig. 4). Interestingly, chemically different odorants clustered together in their response profile. Thus, similar OSNs response profile to chemically diverse odorants might reveal a better understanding of ligand receptor interactions.

### Olfactory coding of structurally similar compounds by *S. calcitrans* OSNs

**Figure 5:**
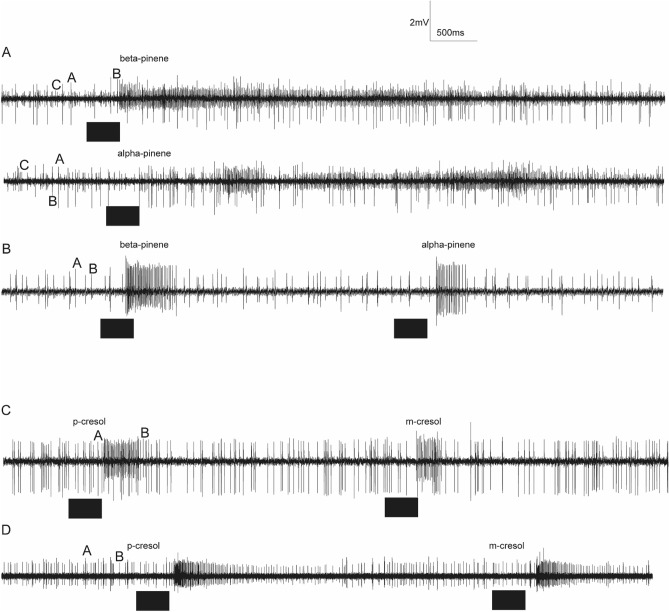
Olfactory coding of isomers by *S. calcitrans* OSNs. (**A**, **B**) Representative SSR traces showing OSNs response for α- and β-pinene from Basiconic sensilla. (**A**) Stimulation with α and β-pinene elicits a strong response from the smaller spiking neuron, indicated by ‘C’ whereas the larger spiking neurons, indicated by ‘A’ and ‘B’ are unaffected by the stimulus (**B**) Stimulation with α and β-pinene elicits a response from the bigger spiking neuron, indicated by ‘A’ (**C**, **D**) representative traces of OSNs response for *m-* and *p*-cresol from basiconic sensilla. Stimulation with p-cresol and m-cresol elicits a strong response from the smaller spiking neuron, indicated by ‘B. Horizontal bar indicates stimulus duration, 0.5 s.

We found that structurally similar compounds or isomers activated the same OSN with different response dynamics. For instance, α- and β-pinene (Fig. 5A,B) excited the same OSN in the same sensillum and p-cresol and m-cresol activated the same OSN in the same sensillum (Fig. 5C,D).

### Behavioural response of *S. calcitrans* to the compounds under field condition

**Figure 6:**
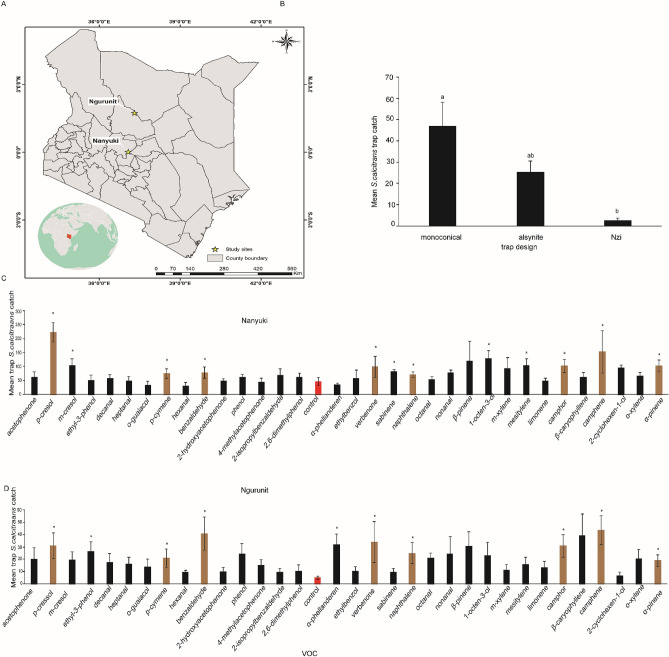
Geographical behavioural response of *S. calcitrans* to VOCs (**A**) Geographical distribution of the two study sites, (**B**) Comparison of three biting fly traps; means followed by different letters are statistically different, ANOVA followed by Tukey’s test. The bar represents SEM (**C**). Mean trap catch of *S. calcitrans* to listed compounds at Nanyuki site. (**D**) Graph showing mean trap catch of *S. calcitrans* to listed compounds at Ngurunit site * shows significant difference between the VOC and control, t-test, P < 0.05. For each treatment n = 6. Light brown the most attractive compounds as compared to control (red bar) at both sites.

To test the behavioural response of VOCs from camel’s sources to *S. calcitrans*, we set and carried out a field trapping experiment at two sites ~ 158 km apart in direct geographic distance (Fig. 6A). Compounds tested are listed in Table 1. We first evaluated the three recommended traps for *S. calcitrans*, unbaited monoconical^23^, alsynite^24^ and Nzi^25^ traps. Our results show that the monoconical and alsynite traps caught more *S. calcitrans* than the Nzi trap, P < 0.05 ANOVA followed by Tukey post Hock test) (Fig. 6B). However, the monoconical trap caught two-fold more *S. calcitrans* (42 ± 12.5) than the alsynite trap (22 ± 5.1) (Fig. 6B). Next, we baited the monoconical traps with 33 camel-derived compounds. VOCs that may be conserved in two or more metabolic products and activated more OSNs were more attractive than those present in one metabolic product as compared to control, P < 0.05, * independent t-test (Fig. 6C,D). *p*-cresol, which is shared between three metabolic products and activated more OSNs, was the most attractive compound at both sites, p < 0.05, t-test. A trap baited with p-cresol caught on average 223 flies/per trap/day at Nanyuki and 32 flies/per trap/day at Ngurunit. Furthermore, *p*-cymene which is shared between the four metabolic products was more attractive than the unbaited control trap to *S. calcitrans* at both sites. Benzaldehyde was present in urine and body odour and attracted more *S. calcitrans* (Fig. 6C,D) than the unbaited control trap at both sites.

**Table 1:** Volatile compounds identified from different camel odour sources and tested in physiological and behavioural assays.

Compound	Camel metabolic source	CAS no.	RT (min)
Acetophenone	Urine, skin/body	98-86-2	12.47
*p*-Cresol	Urine, skin/body, dung	106-44-5	12.74
Ethyl-3-phenol	Urine	620-17-7	14.24
Decanal	Skin/body, urine	112-31-2	14.71
Heptanal	Skin/body	111-71-7	9.11
*o*-Xylene	Skin/body, dung, and breath	95-47-6	8.84
*o*-Guaiacol	Skin/body, urine	90-05-1	12.89
*p*-Cymene	Dung, skin/body, urine and breath	99-87-6	11.66
Hexanal	Skin/body	66-25-1	6.46
Benzaldehyde	Urine, skin/body	100-52-7	10.41
2-Hydroxyacetopheneone	Urine	118-93-4	14.10
Phenol	Urine, skin body, and dung	108-95-2	10.97
4-Methyacetophenone	Urine	122-00-9	14.48
2-Isopropylbenzaldehyde	Urine	122-03-2	15.31
2,6-Dimethylphenol	Urine	576-26-1	13.18
2-Cyclohexen-1-one	Urine	78-59-1	11.30
*α*-Phellandrene	Urine, dung	555-10-2	11.28
Verbenone	Urine, skin/body	1196-01-6	14.91
Sabinene	Urine	3387-41-5	10.69
Naphthalene	Skin/body, breath	91-20-3	14.19
Octanal	Skin/body	124-13-0	11.05
Nonanal	Skin/body, and urine	124-19-6	13.09
β-Pinene	Skin/body, dung	18172-67-3	10.74
1-Octen-3-ol	Skin/body	3391-86-4	10.88
*m*-Xylene	Skin/Body, dung, and breath	108-38-3	8.28
Mesitylene	Skin/body, dung, and breath	108-67-8	10.61
*α*-Pinene	Dung, and skin/body	80-56-8	9.80
Limonene	Dung	5989-27-5	11.75
Camphor	Dung	76-22-2	13.86
*β*-Caryophyllene	Dung, and skin/body	87-44-5	17.78
Camphene	Dung	79-92-5	10.12

However, the aldehydes, hexanal, heptanal, and decanal identified only from body odour when tested, they were less attractive than the unbaited control trap. Similarly, we observed that there was no significant difference between trap captures with the urine-specific volatiles 2-hydroxyacetophenone and 4-methylacetophenone and the unbaited control trap. Interestingly, the behavioural response between structurally similar compounds was different with respect to the trap captures. The same pattern was observed in the electrophysiological responses to these compounds (Figs. 4, and 5). For instance, *p*-cresol attracted significantly more flies than *m*-cresol at both sites (p < 0.05, t-test) (Fig. 6C,D). Likewise, of the two isomers α- and β-pinene, the former attracted more stable flies than the latter compound at both sites compared to the unbaited trap (Fig. 6C,D).

We asked how consistent those attractive odors at Nanyuki site in their attractivity at different site (Ngurunit), and we repeated the same experiment at different places with different flies’ density. When we compared the trap catches between the two sites, 8 compounds retained their attractive effects at both sites brown bars (Fig. 6C,D). These attractive compounds, except verbenone clustered together in their OSN response profile (Fig. 4). However, 12 compounds (Fig. 6C*) attracted more flies than the control at the Nanyuki site, whereas only ten compounds were more attractive at Ngurunit site (Fig. 6D*). However, we found that the fly density at Ngurunit was less than found at the Nanyuki site. Furthermore, we tested the blends constituted from the unique VOCs for each metabolic product, which did not attract a significant number of flies as compared to unbaited control (data not shown).When we correlate the physiological and behavioural responses the attractive VOCs activated more OSNs, for instance, *p*-cresol activated 67% of the targeted OSNs, whereas *p*-cymene, α-pinene, and naphthalene activated 52% of the targeted OSNs.

### Behavioural response of house flies to camel VOCs

**Figure 7:**
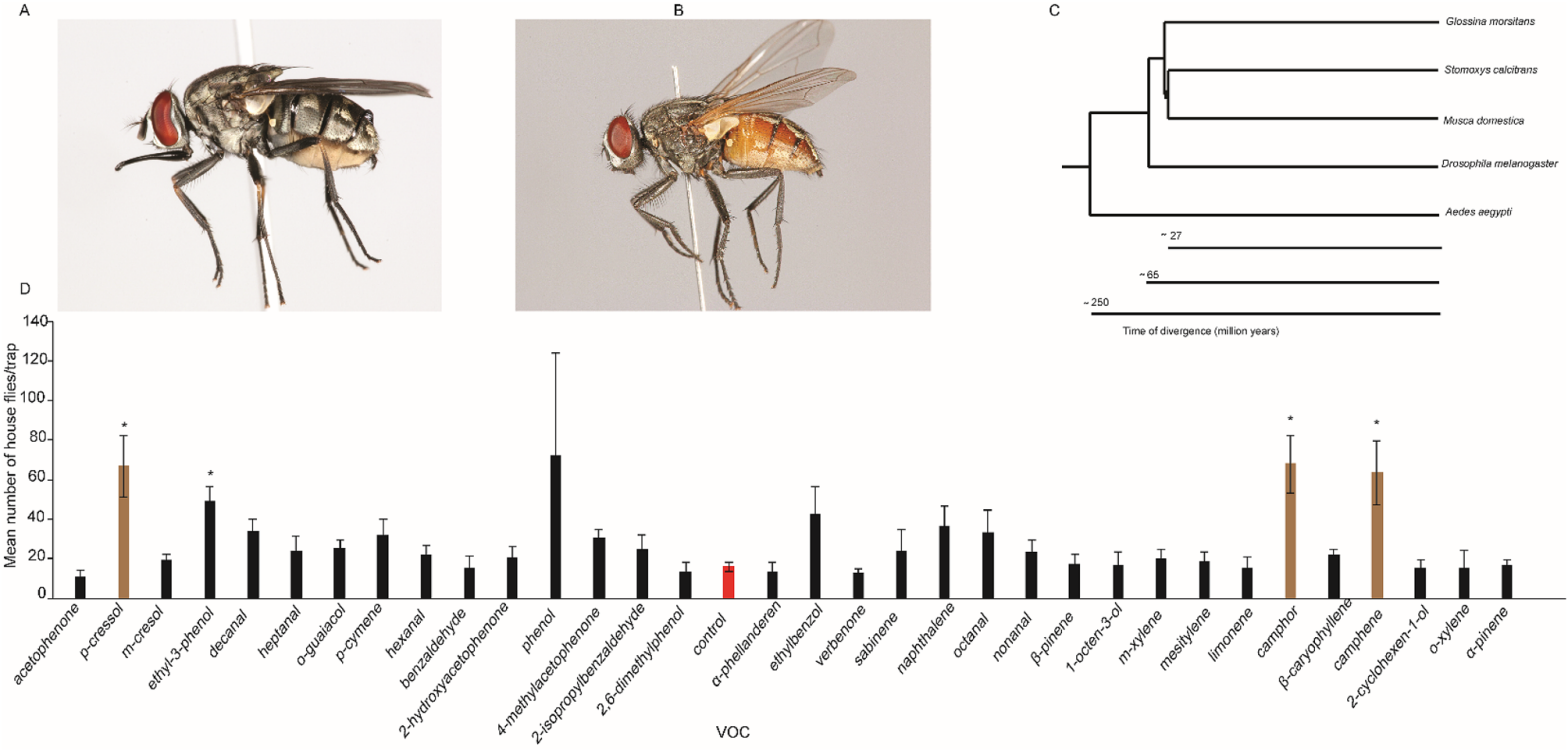
Response of house flies to camel VOCs. (**A**) *S. calcitrans* (stable fly), (**B**) *Musca domestica* (House fly) (**C**) Phylogenetic tree showing the evolutionary relationship of the two species within the order Diptera (**D**) Mean trap catch of house flies to listed compounds under field condition, n = 6. The brown bar shows the most attractive to both stable flies and house flies. Red bar control trap.

We posited whether stable fly (Fig. 7A) and house fly (Fig. 7B), which coexist in the same ecological niche, but diverged ~ 27 million years ago^26^ (Fig. 7C) use the same semiochemicals in identifying their camel host. House flies are opportunistic blood feeders. Analysis of the trap captures with traps baited with *p*-cresol, ethyl-3-phenol, camphene, and camphor, attracted significantly more house flies than the unbaited control trap * shows significant difference between the VOC and control, independent t-test P < 0.05 (Fig. 7D). The three VOCs, except for ethyl-3-phenol were attractive to the stable fly at both sites, showing that the response of these two distinct flies to some of the compounds is conserved. In total, only four VOCs attracted significantly more house flies, as opposed to 10 for stable flies at the same site and time, showing there are also species specific responses to VOCs in each species. House fly data is only from Ngurunit, as the number of house flies in Nanyuki was too low for any meaningful statistical analysis.

## Discussion

Our comparative VOC analysis of the four metabolic products (urine, body, dung, and breath) of the same camel highlighted important aspects of intraspecific diversity of semiochemicals. The different odour sources had distinct volatile profiles both in identity and relative abundance of common VOCs. Our results also demonstrate that each odour source appears to have its own signature chemical bouquet, even though they originated from the same camel. However, we found similarity in the composition of the VOCs between the same odour sources between populations. This indicates that variation in VOCs dependent on each specific metabolic product rather than between camels. The characterization of the VOCs of camel from the four possible metabolic products and comparison of their physiological and behavioural responses in the stable fly provides several insights into host–vector ecological interactions. Although, several chemical ecology studies have investigated different livestock and vector interactions that have enabled identification of attractants, and repellents^17,27,28^ to the best of our knowledge this is the first study to compare the VOCs between metabolic products of the same animal.

The distinct VOCs profiles observed between metabolic products suggest that VOC production in the camel may be driven by specific metabolic processes associated with microbes and tissue-specific enzymes^29^. Unlike animals, most VOCs research has been carried out on plants, but interestingly, the findings in the present are consistent with similar findings found for plants^30,31^. For example, comparative studies in plants have demonstrated variations in metabolite composition within individual plant parts^30,31^. This intra-individual chemodiversity includes all plant parts, from roots to reproductive tissues^32–34^. Similarly, plant part specific VOCs emission have been found in the same plant, whereby stamens, sepal and carpel of the same plant released distinct VOCs^35^. Furthermore, VOC emissions from maritime pine have demonstrated that the pinene complex emissions from branches had a distinctly different chemical signature than pine emissions from the stems of the same trees^36^. Our multidimensional VOCs analysis suggests that a few compounds correlated with the separation of the four metabolic products. For example, heptanal, heptadecane, geranyl acetone, 1-octen-3-ol were only detected in camel body odour. Ethyl-3-phenol, 2-cyclohexen-1-one, 2,6-dimethylphenol, pinocarvone, phenylacetone were detected only in urine odour. Limonene, camphor, β-citronellene, α-bergamotene were detected only in dung odour. Dodecane and 1,2,4-trimethylbenzene were detected in only breath odour, showing that each metabolic product has its own specific signature compounds, and as previously indicated might be associated with the microbiome and tissue -specific enzymes.

A previous study^21^ identified general predictive VOCs for animal dung such as, *p*-cresol, phenol, indole and benzaldehyde. In the present study, *p*-cresol, phenol were detected in the odour of camel feces in trace amounts, but benzaldehyde and indole were not detected indicating that camel feces, which was not included in^21^ has a different predictive volatile. Interestingly, benzaldehyde was detected in both urine and body odour of the camel. Similarly, in previous study^12^ predicted different compounds as constituting the signature odour for camel dung. According to^12^, *p*-cymene was a dominant predictive compound, and interestingly *p*-cymene might be the only compound shared between the four metabolic products of camel detected in significant levels. The variation in abundance between shared compounds between metabolic products might show the variation in the pool of potential VOC odour sources.

Our physiological and behavioural analyses showed that behavioural attraction increased with shared VOCs between metabolic products, followed by activation of more OSNs. For instanc*e, p*-cymene, which activated 52% of the OSNs and shared by the four metabolic products, was more attractive to *S. calcitrans*. Similarly, *p*-cresol shared compound between camel dung, body, and urine, activated 67% of the OSNs, and was more attractive regardless of geography. However, 1-octen-3-ol detected only in body odour, even though it activated 61% of the OSNs, was attractive only at one site where the fly density was high. Such difference in the attractiveness of a given VOC might indicate interaction between the activated OSNs and the recruited glomeruli, which in turn might determine the odour perception. Previous research demonstrated that *p*-cresol elicited a strong response in electrophysiological recordings using antennae of the stable fly which supports our findings^37^. Activation of more OSNs and summation of their response could result in stronger EAG response^37^. Similar, results have been demonstrated in fruit flies, where by shared odours between different fruits attracted more flies^38^. Our results demonstrate that, a large degree of quantitative and qualitative variation occurs in the VOCs in the different metabolic products from the camel. The lack of significant attraction from blends of each metabolic products, might be due to the formulation used to prepare the blends or the spatiotemporal presentation of blend stimuli for the behavioral study. This is often a challenging task requiring volatiles with diverse chemical and physical properties to be presented as a unified stimulus, and necessitates accurate control of the timing, concentration and homogeneity of the odor stream to mimic their natural release rates from the camel.

There are several structurally similar compounds or isomers in the VOCs detected from the camel. How these VOCs code might determine how they are discriminated. When we compare the physiological and behavioural responses between isomers such as *m*-cresol and *p*-cresol, or β-pinene and α-pinene, we found that the two respective isomers were detected by the same OSN but elicited different physiological (Figs. 3, 4, and 5) and behavioural responses (Fig. 6C,D). The response of the two pairs of structurally similar compounds vary, ranging from strong to weak excitation, to strong attraction to neutral response. Similarly, in Drosophila, 25 structurally similar compounds elicited different physiological and behavioural responses, that ranged from strong attraction to repulsion^39^. Likewise, in *Pachnoda marginata,* compounds detected by the same receptor elicited different attraction response from the insect^40,41^. The different behavioural responses elicited by the two structurally similar compounds might be explained by different response from OR repertoire both in terms of response magnitude and response dynamics, that result in a personalized percept beyond the chemistry of the molecule. As previously demonstrated, a small difference in the OSNs response magnitude leads to amplification of a given odourant to produce a large difference in the representation among higher-order neurons^42–44^. However, in other studies chemically similar odours were found to evoke similar physiological and behavioural responses^45,46^, showing a conserved circuit from periphery to the high brain region that organizes information about odour relationships to ultimately support similar perception.

The volatilome comparison between the various odor source from the same animal followed by physiological and behavioural expriments will enable to identify reliable attractant and provides an innovative solution for vector management tools development. The development of resistance to multiple insecticides both in house flies and stable flies^9^, make chemical ecology approaches to identify potent semiochemical-based lures as an alternative option for these vectors management. Such lures could be powerful tools for monitoring, but also for mass trapping of *S. calcitrans* and house flies, which are important pests of livestock and humans^47^.

## Conclusion

Comparative VOCs between different metabolic products followed by olfactory sensory neurons and behavioural response will provide a better host-vector olfactory interaction mechanism to address evolutionary and ecological questions. It can also offer a tool to accelerate the identification of reliable olfactory cues as an attractant for monitoring and control of insect vectors and pests. Our results show that, VOCs shared between metabolic products and which have similar response profile elicited a strong behavioural response under field condition. Thus, the identified compounds can be used to control stable flies and house flies, vectors of animal disease pathogens.

## Materials and methods

### Ethics statement

We collected urine, dung, sweat, and breath from camel within the accordance of the International Centre of Insect Physiology and Ecology’s Institutional Animal Care and Use Committee (IACUC) guidelines. CITI training program, on animals handling and use, was undertaken and certificate acquired prior to the approval of the proposed study by *icipe* IACUC and approved as the following REF: icipe-IACUC-10/2018.1. The odour collection from camels was done with the authorisation of the owner. Herdsmen/women gave their consent for their animal sampling after explaining the objectives of the study. No samples other than those for odour collection procedures were collected.

### Trapping of VOCs from odour sources

#### Camel

Since emissions of VOCs can vary with age, sex and diet of an organism, we chose adult female camels 8 years old for the study. The camels were free-ranged fed on natural vegetation in the same village, where various acacia species with open grass are the most dominant plant species for browsing. All camels selected for the study were assumed to be healthy.

### Trapping of VOCs from odour sources

#### Urine

Fresh urine (500 ml) was collected from five 8 year-old-female camels early in the morning between 6 and 7 a.m. in a plastic bucket and transferred to a 1 l glass jar. Volatiles were collected from the urine for 12 h using a dynamic headspace odour collection technique comprised of a portable vacuum pump, Porapak™ Type Q adsorbent (Sigma scientific USA). The trapped odours were eluted with 200 µl hexane. Clean air was pushed with 2.5 l/min while the vacuum or pull was set at 2 l/min using a Sigma portable asymmetric volatile collection pump (www.sigmascientifc.com).

#### Dung

Similarly, fresh dung was collected from the same camel and kept in a cool box until 500 g of dung was collected. Volatiles were collected and eluted as described in (a) above.

#### Body

From the same camel, body odours were trapped on clean new cotton odourless dress by rubbing all over the body for 10 min. The swab was placed again in a clean glass jar for headspace trapping as described in (a). The clean cotton cloth odour trapped similarly was used as a control.

#### Breath

The camel was restrained from moving with the hands by two people. Teflon bags were used to cover the mouth and nose to concentrate the volatiles coming out from the breath. This approach allowed for isolation of environmental odours. Porapak Q adsorbent attached to the Sigma portable asymmetric volatile collection pump with the Teflon tube was place in front of the mouth and nose alternatively. VOCs were trapped for maximum of 30 min in 15 min intervals to avoid any discomfort to the camel. The extract was eluted the same way with 200 µl of hexane as described for other odour sources.

### GC–MS analysis

We used a gas chromatograph coupled to a mass spectrometer (GC–MS; HP 6890 GC and 5975 MS; Agilent Technologies, Palo Alto, CA, USA) to analyze the collected volatiles in the electron impact at 70 eV. Helium was used as the carrier gas at an average linear flow rate of 35 cm/s. An autosampler (Agilent Technologies) was used to inject 1 μl of each sample into the GC–MS on a nonpolar capillary HP-5 column. Injections of the volatile extracts were conducted in a splitless injector at 220 °C. The oven temperature was programmed: 35 °C for 5 min and then increased by 10 °C/min to a final temperature of 280 °C, and held at this temperature for 10 min. Mass spectra and retention times of volatiles were compared (Kovat’s indices) with their commercial standards and library database spectra using the NIST mass spectral program (ver. 2.0), Pherobase (http://www.pherobase.com) and the NIST web book (http://webbook.nist.gov/chemistry). When sample compounds were available, we co-injected these standards and compared their spectra and retention times of the predicted compounds to confirm their identities.

### Electrophysiological response characterization using single sensillum recording (SSR)

The single sensillum recording (SSR) is a form of extracellular electrophysiology, where action potentials generated by OSN(s) within single sensilla found on the insect antenna can be measured by an electrode in contact with the extracellular receptor lymph. We have chosen SSR as it is very effective extracellular recording technique for mapping the receptive range of the olfactory sensory neurons housed within the sensilla^48,49^. The SSR procedure was performed as described previously^50–52^. Four to six days old adult flies were immobilized in pipette tips (1000 µl), and the third antennal segment was mounted on microscopic slides and immobilized with glass electrodes by pushing it on the coverslip. Basiconic Sensilla were localized under an Olympus binocular microscope at 1000× magnification, and the extracellular signals originating from the OSNs were measured by inserting a tungsten wire electrode into the base of a sensillum. The reference electrode was inserted into the eye. Signals were amplified (10×; Syntech Universal AC/DC Probe; sampled (10,667 samples/s) and filtered (100–3000 Hz with 50/60-Hz suppression) via a USB IDAC connection to a computer. Only a single recording was made from a single fly to avoid OSN desensitization. Action potentials were extracted using Syntech Auto Spike 32 software (Syntech, http://www.ockenfels-syntech.com). The activity of co-located OSNs in single sensilla was differentiated based on differences in their spike amplitude. Neuron activities were analyzed 1 s before and after, the 0.5 s odour stimulation by Syntech stimulus delivery. Responses from individual neurons were calculated as the number of spikes during stimulation minus the spontaneous activity before the stimulation. For SSR recording, test compounds stimuli were randomized. The odors dissolved in hexane and 10 µl was loaded on 1 cm diameter filter paper inserted in glass Pasteur pipette. Humidified air from gas washing bottle filled with distilled water, was blown on the mounted antenna at 20 cm/s.

### Field testing of selected volatile organic compounds

Synthetic compounds (Table 1) were purchased from (Sigma Aldrich, Germany). We tested the attractiveness of the listed compounds under field conditions at two different sites; Mpala Ranch, Laikipia County in central Kenya (Fig. 5A; 00° 23′ 26.98″ N, 036° 52′ 14.98″ E) and Ngurunit, Marsabit County (N 01° 0.74′, E 037.29′). These regions are characterized by arid and semi-arid savannah vegetation in which *S. calcitrans* is one of the most abundant biting flies^2^. First, we carried out a simple trap comparison between three existing traps recommended for *S. calcitrans*; monoconical trap^23^, alsynite^24^ and Nzi^25^ with all three unbaited in a randomized complete block design (n = 5). Next, we compared the attraction of the compounds to the flies as previously described^12,53^. Each trap was baited with 2 ml of neat synthetic standard of each listed compound (Table 1). All treatments in six replicates were placed 100 m apart and each block separated by 500 m from each other. Each VOC was assigned to a given position randomly using a simple randomization technique. Each VOC was dispensed from 4 ml glass vial with 5 holes of 2 mm diameter each. A cotton dental roll (10 × 38 mm; Shanghai Dochem Industries Co. Ltd.) was inserted inside the vial as a dispenser. The vial was tightened on the metal pole with a wire, 0.5 m above the ground^12^. Unbaited trap was used as a control.

### Statistical analyses

To determine differences in VOCs released by the different odour sources, chromatograms were assessed using the relative abundance data in Past Version 3.02. We included VOCs detected in at least 2 out of 5 camels, with relative abundance ≥ 0.15% in the analysis. We assessed whether volatile composition was associated to a specific metabolic product and among camel populations using non-metric multidimensional scaling (NMDS) ordination analysis^35,54^. NMDS allowed as to visualize the similarities of VOCs between metabolic products. It also allowed comparison of not only quantitative data, but also data sets that contain compounds that are non-normally distributed and/or categorical (i.e. zeros are prevalent). The chemical profiles of different metabolic product emissions were compared by one-way ANOSIM using Bray–Curtis dissimilarity matrix^54^. Techniques such as NMDS and ANOSIM SIMPER analyses of pasta version 3.02 were used to determine the relative contribution of different compounds to the dissimilarity between the headspace of the four metabolic products VOCs^54^. Multivariable statistical analysis on OSN response (i.e., cluster analysis) was performed using PAST version 3.02. The *S. calcitrans* catch between the three traps was compared using one-way ANOVA. *S. calcitrans* behavioural response under field condition was compared using independent t-test. Individual VOC was tested against control. Prism (v23, IBM, New York, NY, USA) was used for data analysis.

## Supplementary Information

Supplementary Table.

## Data Availability

All the primary experimental data used in compiling this paper are included in the figures and list of VOCs identified in the four metabolic products in the Supplemental Table.
