# Supplementary material for: Shared volatile organic compounds between camel metabolic products elicits strong Stomoxys calcitrans attraction
Source: Sci Rep. 2020 Dec 8;10:21454. doi: 10.1038/s41598-020-78495-9 (PMC7722739; doi:10.1038/s41598-020-78495-9)
Supplement: Supplementary file 1 — Supplementary Table. [file 41598_2020_78495_MOESM1_ESM.docx]

**Merid Negash Getahun^1^*, Peter Ahuya^1^, John Ngiela, Abel Orone^1#^, Daniel Masiga^1^**

**and Baldwyn Torto^1^**

1. International Centre of Insect Physiology and Ecology(icipe), P.O. Box 30772‑00100, Nairobi, Kenya

**#** current address, Kenya Agricultural & Livestock Research Organization (KALRO)

Biotechnology Research Institute, Nairobi Kenya.

*mgetahun@icipe.org

**Shared volatile organic compounds between camel metabolic products elicits strong vector attraction by activating most olfactory sensory neurons**

Supplemental table. List of volatile organic compounds (VOCs) identified in the four metabolic products
